# Supplementary material for: Longitudinal Analysis of SARS-CoV-2-Specific Cellular and Humoral Immune Responses and Breakthrough Infection following BNT162b2/BNT162b2/BNT162b2 and ChAdOx1/ChAdOx1/BNT162b2 Vaccination: A Prospective Cohort in Naive Healthcare Workers
Source: Vaccines (Basel). 2023 Oct 19;11(10):1613. doi: 10.3390/vaccines11101613 (PMC10610978; doi:10.3390/vaccines11101613)
Supplement: Supplementary file 1 [file vaccines-11-01613-s001.zip › vaccines-2642283-supplementary.pdf]

Supplementary Materials

Table S1. Mutations in spike protein of the B.1.1.529/BA.1 BA.2 and BA. 5 lineages (Omicron variant) compared to the Wuhan variant.

Wuhan Wild-Type		Omicron Variants		
Sequence position	Amino acid	B.1.1.529/BA.1	B.1.1.529/BA.2	B.1.1.529/BA.5
19	T19		T19I	T19I
24	L24		L24deletion	L24deletion
25	P25		P25deletion	P25deletion
26	P26		P26deletion	P26deletion
27	A27		A27S	A27S
67	A67	A67V		
69	H69	H69deletion		H69deletion
70	V70	V70deletion		V70deletion
95	T95	T95I		
142	G142	G142D	G142D	G142D
143	V143	V143deletion		
144	Y144	Y144deletion		
145	Y145	Y145deletion		
211	N211	N211deletion		
212	L212	L212I		
213	V213		V213G	V213G
214		Insertion214EPE		
339	G339	G339D	G339D	G339D
371	S371	S371L	S371F	S371F
373	S373	S373P	S373P	S373P
375	S375	S375F	S375F	S375F
376	T376		T376A	T376A
405	D405		D405N	D405N
408	R408		R408S	R408S
417	K417	K417N	K417N	K417N
440	N440	N440K	N440K	N440K
446	G446	G446S		
452				L452R
477	S477	S477N	S477N	S477N
478	T478	T478K	T478K	T478K
484	E484	E484A	E484A	E484A
486				F486V
493	Q493	Q493R	Q493R	
496	G496	G496S		
498	Q498	Q498R	Q498R	Q498R
501	N501	N501Y	N501Y	N501Y
505	Y505	Y505H	Y505H	Y505H
547	T547	T547K		
614	D614	D614G	D614G	D614G
655	H655	H655Y	H655Y	H655Y
679	N679	N679K	N679K	N679K
681	P681	P681H	P681H	P681H
764	N764	N764K	N764K	N764K
796	D796	D796Y	D796Y	D796Y
856	N856	N856K		
954	Q954	Q954H	Q954H	Q954H
969	N969	N969K	N969K	N969K
981	L981	L981F		

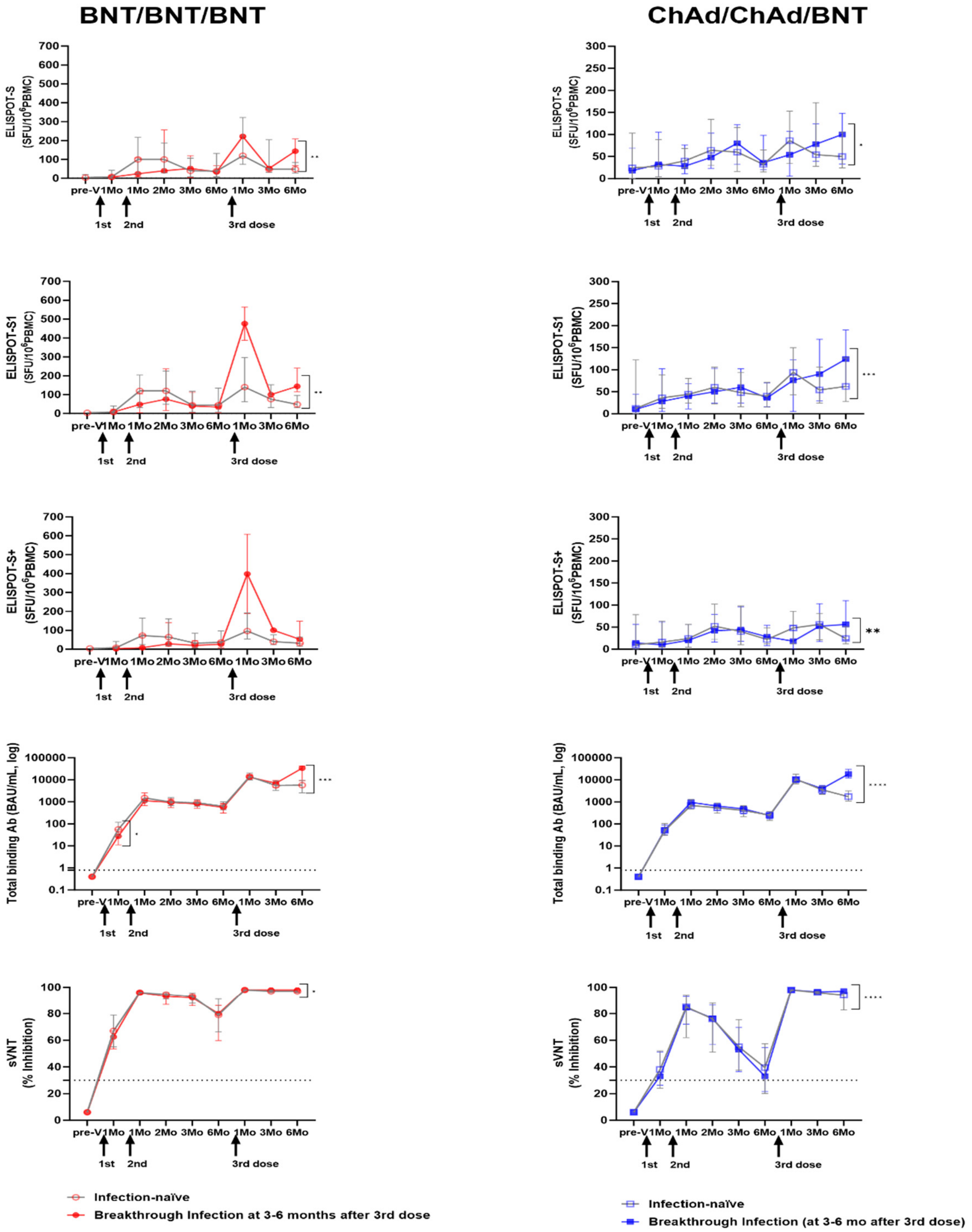

Figure S1. Comparison of longitudinal immune responses between infection-naïve and breakthrough infection participants. ELISPOT results for S, S1 and S+ peptides and humoral response measured by total binding antibody and neutralizing activity levels, up to 6 months after the 3rd dose in the BNT group (A) and the ChAd group (B). Within each group, the immune response was compared between infection-naïve participants and those who developed breakthrough infections 3 to 6 months after the third dose.

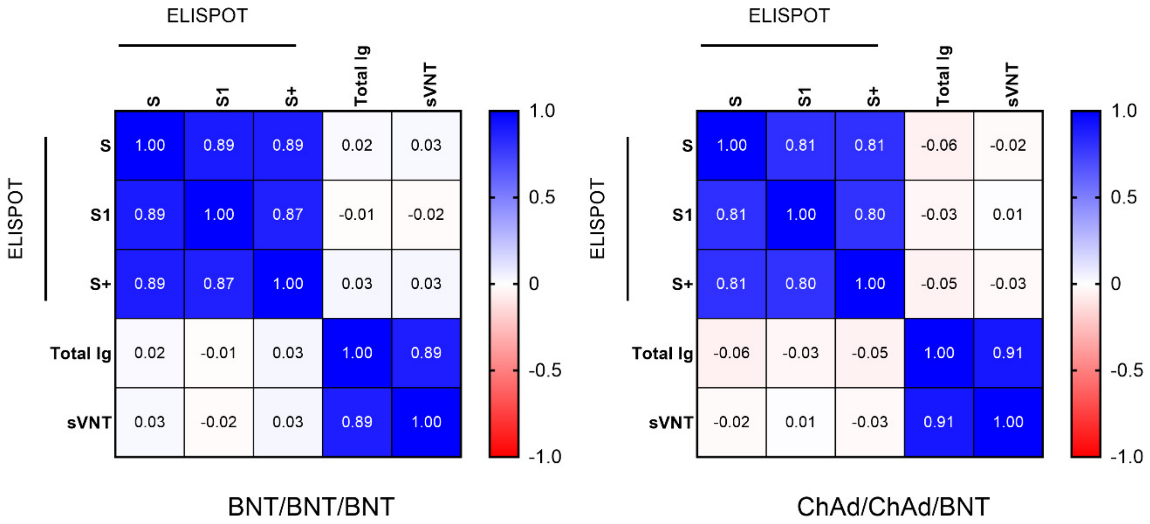

Figure S2. Correlation between SARS-CoV-2-Spike specific cellular response measured by ELISPOT, and humoral responses assessed by total immunoglobulin and neutralizing antibody assay (sVNT) in the BNT/BNT/BNT (A) and the ChAd/ChAd/BNT (B) groups.

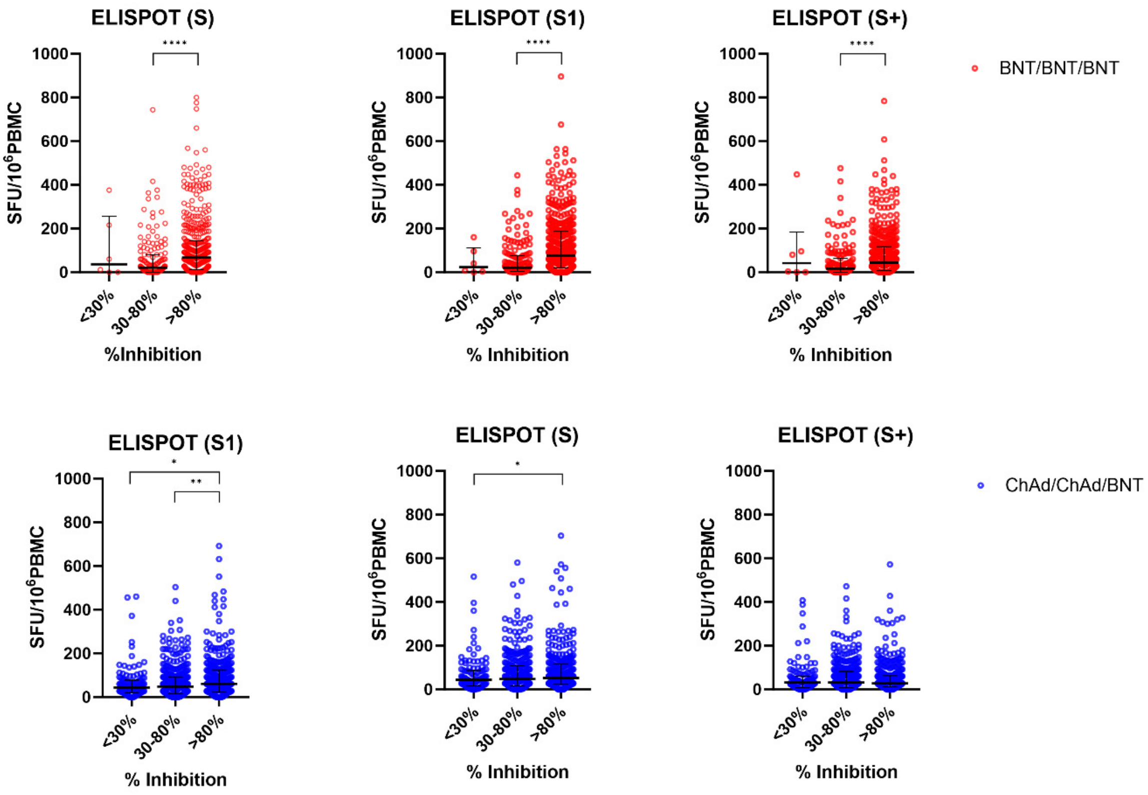

Figure S3. Comparison of ELISPOT results according to the classified sVNT results into three grades (strong: >80%, moderate: 30–80%, weak: <30%).
